# Reference Values for Habitual and Fast Gait Speed in Singapore Adults Aged 21 to 80

**DOI:** 10.3390/jcm13123507

**Published:** 2024-06-15

**Authors:** Mingxing Yang, Leik Yu Leung, Zhi Yan Lim, Richmond W. Ang, Ho Man Ip, Xin Qian Lee, Kellee Y. Lim, Li Ching Teoh, Meredith T. Yeung

**Affiliations:** 1 Allied Health, Singhealth Polyclinic, Singapore 150167, Singapore; yang.ming.xing@singhealth.com.sg; 2 Health and Social Sciences Cluster, Singapore Institute of Technology, Singapore 138683, Singapore

**Keywords:** 4 m gait speed test, Singapore, normal healthy population, functional status, normal reference values

## Abstract

**Objectives**: Gait speed indicates the individual’s functional status and predicts overall health. This study aims to determine (1) the intra- and inter-rater and test–retest reliability of the dynamic 4 m gait speed test protocol; (2) establish the normative reference values of habitual and fast gait speeds in community-dwelling healthy Singaporean adults aged 21 to 80; and (3) explore the association of age, gender, height, weight, and body mass index (BMI) on gait speed. **Methods**: This prospective cross-sectional study recruited healthy ambulatory community-dwelling Singaporeans aged 21 to 80 who could ambulate independently without aid. Participants were excluded if they required walking aids; were pregnant; or had physical, medical, or cognitive conditions that may affect gait. Each participant completed at least two habitual and fast gait speed test trials via a 4 m walkway with a dynamic start. The data were analysed by descriptive statistics, the Mann–Whitney test, the Spearman coefficient, and the interclass correlation coefficient (ICC). **Results**: In total, 178 males and 201 females were included in the data analysis. The median age was 45.0 years [interquartile range (IQR) 26.2–59.0], and the median height was 1.64 metres (m) (IQR 1.58–1.70). The median habitual gait speed was 1.08 metre/second (m/s) (IQR 0.97–1.22), and the fast gait speed was 1.55 m/s (IQR 1.40–1.70). The ICC for reliability ranged from 0.84 to 0.99, indicating that the 4 m gait speed test had good-to-excellent reliability. **Conclusions**: Gait speeds were not influenced by gender but declined with age advancement. Age and height and age and BMI were weakly correlated to habitual and fast gait speed, respectively. We established the norm values for the 4 m gait speeds in Singapore and proved it to be a reliable gait speed assessment ready for immediate community applications.

## 1. Introduction

Gait has been described as a “functional” vital sign [1] and an important indicator of physical function with high validity, reliability, sensitivity, and specificity that can predict overall health [2], mortality risk [3], rehabilitative prognosis, indication of fear of falls [4,5,6], and quality of life [7,8]. Gait speed is a straightforward, cost-effective, and clinically relevant assessment [3]. Slow gait speed has been used as an indicator of sarcopenia and increased mortality risk [6,9,10]. Establishing normative values for a reference population is imperative in efforts to benchmark individual performance against a specific population. Previous studies have established that gait speed is influenced by age, gender, and height. Established studies commonly provide age- and gender-specific gait speed values, but these studies often focus primarily on the speed range of older adults [11,12,13]. None have included the reference range of younger adults except for disease-specific studies in recent years. While gait speed performance seems age-related [14], it also appears to be influenced by multiple factors based on available studies. The utilisation of gait speed assessment as a tool for identifying individuals susceptible to health deterioration and tracking functional improvement towards enhanced functionality and life quality may hold significant implications [15], and this can be applicable even in the younger adult population.

While most of the recent studies have established norms for habitual gait speed, few have provided references for fast gait speed [13,16,17,18]. Studies suggest that habitual and maximum gait speeds are excellent indicators of functional dependency among the Japanese population over other performance-based assessments such as hand-grip strength and the single-leg stand test [19]. Furthermore, fast gait speed provides information about the functional reserve capacity of individuals. It is a more challenging and differentiating test than habitual gait speed while maintaining the same test construct. Some further argue that habitual gait speed is more subjective than fast gait speed, resulting in a lower test–retest reproducibility [20]. The choice of distance for the gait speed test can depend on various factors, each with its own benefit. For example, the 10 m gait speed test has demonstrated excellent reliability in healthy and diseased populations. The interclass correlation (ICC) of the 10 m gait speed test was found to be 0.98 [21]. However, the availability of the long walkway in clinical settings can be a challenge. By contrast, the shorter walkway required for the 4 m gait speed test may be more feasible given the minimal space required; yet, some reports suggest it has a lower agreement when compared to the 10 m gait speed test [17,22]. 

To date, there are only three studies [12,18,23] that explored the gait speed of residents in Singapore, with two reporting a more comprehensive age range of 21 to 80 [18,23] and only one reported fast gait speed [18], via the 6 m and 10 m gait speed tests. No available studies investigated the psychometric properties of the 4 m gait speed test. The field of gait speed investigation remains underexplored. Thus, this study aims to determine (1) the intra- and inter-rater and test–retest reliability of the dynamic 4 m gait speed test protocol; (2) establish the normative reference values of habitual and fast gait speeds in community-dwelling healthy Singaporean adults aged 21 to 80; and (3) establish the correlations between gait speed and anthropometric variables, such as age, gender, height, weight, and body mass index (BMI).

## 2. Materials and Methods

### 2.1. Study Design and Ethical Approval

This prospective cross-sectional study included ambulatory community-dwelling Singaporean adults recruited via convenience sampling between June 2021 and January 2023. The University Institutional Review Board approved the study (IRB approval number 2021051). Participants provided written informed consent before data collection; only anonymised data were used during data analysis. No access to information that could identify participants was necessary after data collection for data analysis, and this report does not contain any personal information.

### 2.2. Participants

Participants were recruited conveniently with pamphlets and social media based on the following inclusion criteria: They were community-dwelling Singaporean residents aged 21 to 80, ambulated independently, did not require a walking aid, and could provide informed consent. Participants were excluded if they required walking aids that may artificially enhance gait speed; were pregnant with a potentially altered centre of gravity influencing their gait pattern; had uncontrolled medical conditions and/or cognitive impairments and/or any physical conditions, such as pain, discomfort, joint problems or muscle weakness that may affect gait; or could not provide consent. Recruitment and data collection were carried out in various residential districts across Singapore. The sample size was calculated based on a 95% confidence interval, a margin of error of 5%, and an estimated population proportion of 50%, following the common standard in scientific research for high accuracy, balanced precision and feasibility, and maximum variability. Given these parameters, a sample size of 385 was determined adequate to represent the target population size of 6 million in Singapore [21]. Quota sampling was employed to ensure sufficient numbers for statistical analysis. This non-probability sampling technique is beneficial in this cross-sectional study, allowing for specific subgroups to be adequately represented. In this study, a minimum of 20 participants per age group was targeted to ensure that the sample is representative of the various age groups to increase the study’s external validity.

### 2.3. The 4 m Gait Speed Test Protocol

**Figure 1:**
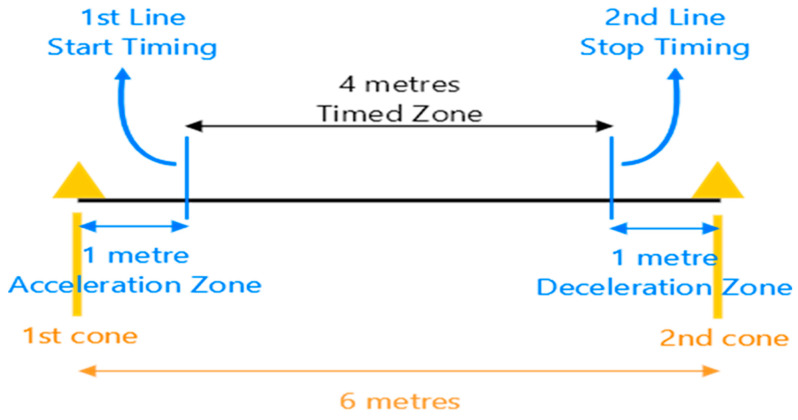
Setup of the 4 m gait speed test.

Participants were instructed to wear comfortable clothes and walking shoes. Figure 1 illustrates the setup of the 4 m gait speed test. The test path was a 6 m long levelled and non-slippery ground path, with a timed 4 m zone and a 1 m acceleration and deceleration zones at the ends [24]. Cones were placed on each end of the path, and two lines marked the start and end of the 4 m walkway. Participants were instructed to walk from one cone to the next without slowing down after hearing the command “3, 2, 1, go!”. The Investigator would start the stopwatch when the first foot passed the start line and stop when both feet passed the second line. Participants were asked to perform trial attempts (habitual and fast pace) during orientation and performed the test at habitual and fast speeds twice each on separate occasions. Habitual speed was defined as their usual walking pace. Participants were told to walk as fast as they could without running for the fast gait speed data collection. The time taken, in seconds, was recorded manually using a digital stopwatch. Gait speed was calculated using 4 metres divided by the time recorded to obtain the gait speed in seconds [metres per second (m/s)]. The two attempts of the habitual and fast speed tests performed separately established the test–retest reliability. The average of the two habitual and fast gait speeds established the normative reference values of the gait speed tests, respectively.

### 2.4. Data Collection

Researchers proficient in conducting field walking tests collected the data. Before the gait speed test, the participants’ age, gender, height (Seca 213 portable stadiometer), and weight (Omron digital weight scale, HN-286) were obtained. BMI was calculated using the standard formula [25]. A pilot feasibility study with 34 participants (n = 34) followed the same 4 m gait speed test protocol [digital stopwatch and 6 m walking track (4 m zone and 1 m acceleration and deceleration zones at the ends)] before the full-scale research to prepare the investigators for logistic, administrative, and procedural requirements. The same inclusion and exclusion criteria were applied to both trial and full protocols. The intra- and inter-rater reliability of the gait speed tests was also determined during the pilot trial. The pilot trial participants performed the two gait speed tests thrice based on random allocation on different occasions.

### 2.5. Statistical Analysis

The data was analysed with GraphPad Prism Version 8.4.3 (GraphPad Software, San Diego, CA, USA); *p* < 0.05 was considered statistically significant. Participants’ demographic data were examined for normal distribution using the Kolmogorov–Smirnov test. Descriptive statistics were used to analyse the central tendency and dispersion, dataset position using median, interquartile range (IQR), and 95% confidence interval (95% CI) of the median. The Mann–Whitney U Test was used to compare variables between genders. The interclass correlation coefficient (ICC) was used to evaluate intra-rater, inter-rater, and test–retest reliability. Spearman rho coefficient (r) determined the correlation between variables. The R Shiny package version 4.4.0 visualised the reference values in an interactive application [26]. To elucidate the influence of age on the correlation between height and gait speeds, we graphically represented the forecasted median gait speeds across various ages. These were specifically for the 10th, 25th, 50th, 75th, and 90th percentile values of gait speed across different ages, corresponding to the gender-specific median height. This approach facilitated a comprehensive understanding of the age-dependent relationship between height and gait speed.

## 3. Results

### 3.1. Reliability

Table 1 presents the reliability of the tests. The ICC of intra- and inter-rater reliability and test–retest reliability ranged from 0.84 to 0.99, indicating good-to-excellent reliability of the 4 m gait speed test protocol [27]. 

### 3.2. Participant Characteristics and Gait SPEED Reference Values

**Figure 2:**
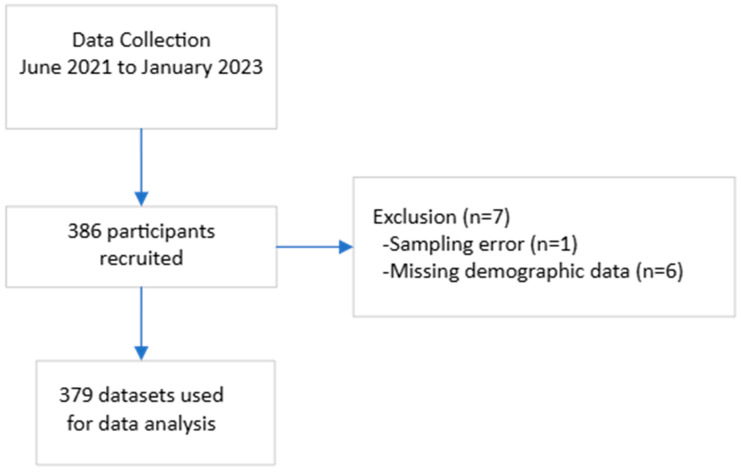
Participant recruitment process.

**Figure 3:**
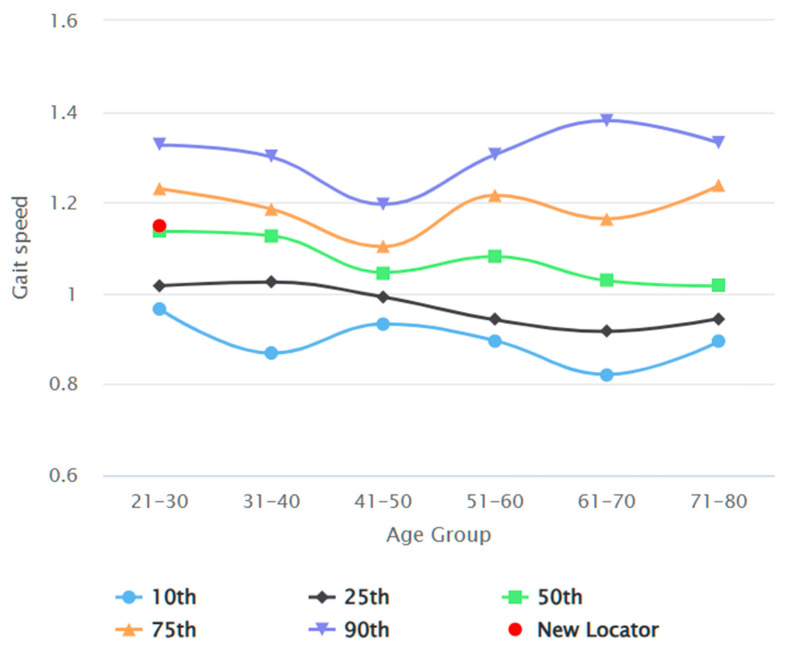
Male habitual gait speed (m/s) against age (years).

**Figure 4:**
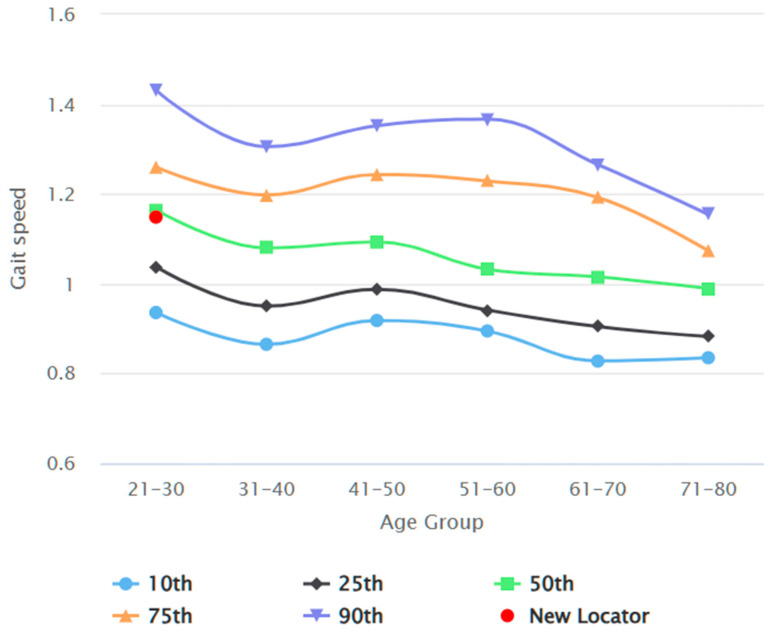
Female habitual gait speed (m/s) against age (years).

**Figure 5:**
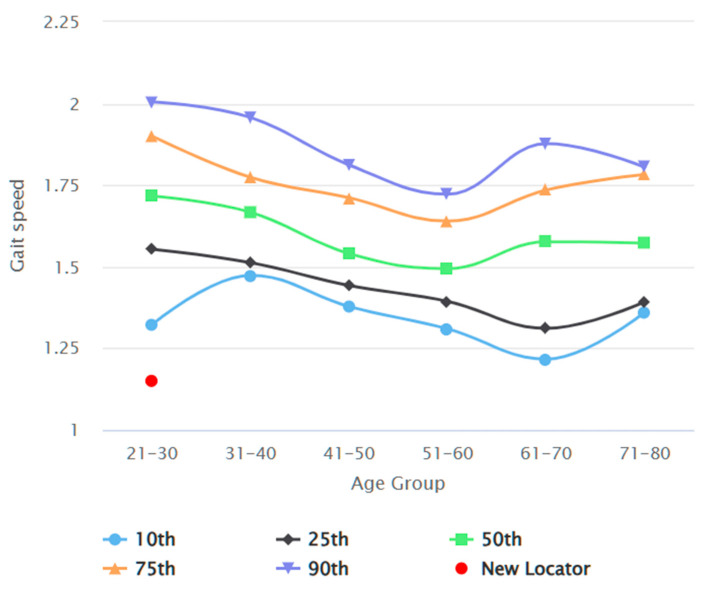
Male fast gait speed (m/s) against age (years).

**Figure 6:**
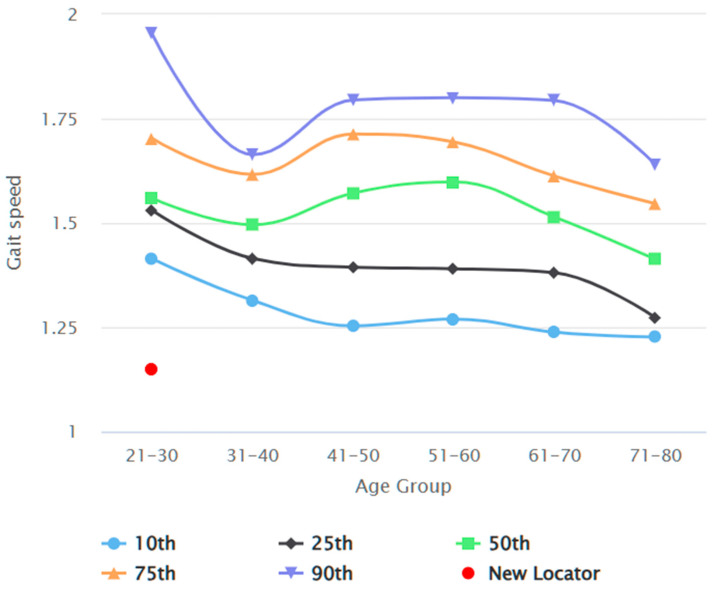
Female fast gait speed (m/s) against age (years).

Figure 2 depicts the participant recruitment process. In total, 386 participants were recruited by the end of data collection. Seven datasets were excluded from data analysis due to sampling error and missing demographics. Table 2 reports the demographics of the study participants, comparing common anthropometric variables between genders. Datasets of 178 male and 201 female participants were analysed. The average habitual gait speed median was 1.08 m/s (IQR 0.97 to 1.22), and the average fast gait speed was 1.55 m/s (IQR 1.40 to 1.70). The differences between gait test 1 and gait test 2 were 0.00 (IQR −0.50 to 0.05) and 0.01 (IQR −0.06 to 0.06), respectively, for habitual and fast gait speeds, indicating a negligible learning effect. There was no statistically significant difference in any of the gait speed results between male and female participants. Table 3 elaborates on the gait speeds split by gender and stratified by age, height, and BMI. Habitual and fast gait speeds decreased with the increase in age across both genders. Taller individuals achieved higher gait speeds, while individuals with more optimal BMI (Groups 18.5–22.9 kg/m^2^ and 23.0–24.9 kg/m^2^) recorded higher fast gait speeds. The gait speed reference values are shown in Figure 3, Figure 4, Figure 5 and Figure 6. Additional reference values for this study are available at https://singaporegaitspeedreferences.shinyapps.io/Gait_Speed/ (established since 15 June 2024). This graphical representation allows for numerical and visual comparison of an individual’s habitual and fast gait speeds with the reference values.

### 3.3. Relationship between Gait Speeds and Variables

Table 4 shows the correlation between gait speeds, demographics, and anthropometric variables. Age (r = −0.24; *p* < 0.001) and BMI (r = −0.1; *p* = 0.043) weakly correlated with habitual gait speed, while age (r = −0.19; *p* = 0.002) and height (r = 0.14; *p* = 0.023) demonstrated similarly weak correlations with fast gait speed.

## 4. Discussion

This study provides clinically valuable normative references on habitual and fast gait speed values for Singaporean community-dwelling adults aged 21 to 80. The proposed dynamic 4 m gait speed test protocol with manual timekeeping demonstrated good-to-excellent reliability (Table 1). A recent systematic review explored the intra-rater reliability for the habitual gait speed test, with an ICC range of 0.72 to 0.98, and the fast speed gait test was reported to have an ICC range of 0.77 to 0.98 [28]. The ICC for inter-rater reliability ranged from 0.79 to 0.95 and 0.98 for habitual and fast gait speed tests, respectively. This study’s results corroborate the gait speed test’s high reliability. Several authors showed that including acceleration and deceleration zones decreases the variability in gait speed and could ensure a more accurate measurement [2,29]. Furthermore, it has been confirmed that gait speed is significantly slower when using a static start [30,31]. There is currently no established standardised distance or type of walkway, and it is not known whether using automatic timers is superior in measuring gait speeds. Therefore, this study’s gait speed test protocol incorporated the 1 m acceleration and deceleration zones in our testing protocol. Our results verified that this simple, low-cost setup provides an excellent alternative in community settings in which sophisticated equipment, such as the GAITRite walkway, may not be available due to limitation of resources. Additionally, an earlier study reported that the manual stopwatch–footfall count method is comparable to a smart system [32], and our results are well aligned with earlier studies [32,33]. 

Our findings are consistent with the established trend of decreasing habitual gait speed [11,23] and fast speed [13] with increasing age. Similarly, the age-related gait speed decline was marked in midlife and further accelerated in the geriatric age groups [13,14]. Gender did not significantly influence gait speeds (Table 2), even with further comparisons made with age, height, and BMI stratifications (Table 3), except in the fast gait speed test between genders of the 31–40 age group (*p* = 0.01). The current study reported that age and height impacted fast gait speed, while age and BMI most affected habitual gait speed. Notably, the habitual gait speed of male participants peaked with the overweight group [BMI 23.0 to 24.9 kg/m^2^; 1.11 m/s (0.97 to 1.24)], and that of female participants peaked with underweight [BMI < 18.5 kg/m^2^; 1.10 m/s (1.02 to 1.25)] and normal weight groups [BMI 18.5–22.9 kg/m^2^; 1.10 m/s (0.96 to 1.28)]. In comparison, the fast gait speed peaked with those weighing normal for both genders. One possible reason could be the decreased muscle mass in the underweight group, leading to suboptimal muscle torque and therefore the inability to produce a higher walking speed [34,35]. By contrast, the overweight group would have to generate more work to overcome the increase in body mass, leading to slower walking speed. However, this could not explain the findings of the habitual gait speeds in the male overweight group. Therefore, we further postulate that this may be an inference from the different body composition between fat and muscle masses despite the eventual increase in BMI [36]. We hypothesised that the male overweight group could consist of individuals with higher muscle mass (muscular obesity) and, thus, recorded with high habitual gait speed. Unfortunately, the data collection did not include the body composition profiles. Therefore, the premise of this justification should be interpreted with caution. Habitual and fast gait speeds increased with height, likely influenced by longer stride length attributable to increased leg length. This explanation could further explain why height is a statistically significant variable correlated with fast gait speed (Table 4), enabling a higher speed, particularly when participants were instructed to walk as fast as they could.

### 4.1. Comparison to the Literature

The available gait speed references varied across studies, possibly depending on differences in the inclusion criteria, such as the demographic of the participants, sample size, and test formats (for example, 4 m, 6 m, or 10 m gait speed tests). Static-start versus dynamic protocol or the exclusion of individuals with comorbidities could lead to a highly selective and non-representative population, resulting in varied reference ranges. Thus, this study adopted relatively loose inclusion and exclusion criteria, as we believe the results could be more relevant from the primary care perspective by being less stringent in excluding individuals with medical conditions.

The length of the walkway could influence the measured gait speeds. Pua et al. (2022) used the dynamic 10 m gait test protocol and reported that the habitual gait speed was 1.34 m/s [standard deviation (SD) 0.23] [12]. By contrast, Lau et al. (2020) reported a gait speed of 1.06 m/s (SD 0.20) and 1.07 m/s (SD 0.19) in male and female habitual gait speeds, respectively, with the 6 m GAITRite walkway system [23]. One possible explanation could be due to a more extended acceleration zone, longer walkway, and a generally fitter population since participants were recruited from fitness programmes. However, the difference in average habitual gait speed reported by Pua et al. (2022) [12] compared to this study is beyond the suggested minimal clinically important difference of 0.1 m/s [37]. The established 1.08 m/s (0.97 to 1.22) (Table 1) average habitual gait speed from the current study is in good agreement with Lau et al. (2020) [23]; these two studies examined the habitual gait speeds for the Singapore population within a similar age range. In other global studies, habitual gait speed was reported to be 1.20 m/s (SD 0.20) in a large cohort study in the Netherlands using a 5.8 m GAITRite Platinum system consisting of a 4.9 m active measurement area [11]. Similar results were reported by Santo et al. (2023) [13] in France, indicating that habitual gait speed was 1.20 m/s and 1.16 m/s for male and female participants in a large-scale Constance Study that investigated gait speed via the MicroGate system with two photoelectric cells placed 3m apart [13]. Our results can draw good parallels with Santo et al. (2023) [13] and Dommershuijsen et al. (2021) [11] after taking into consideration that Asians have been found to have slower gait speed [38] given the generally more diminutive physical stature [39] and lower muscle mass [40].

Upon further comparison with the fast gait speed study reported by Abdul Jabbar et al. (2021) [18], it was found that our participants established lower gait speed with age-matched evaluations. One plausible explanation could be the difference in the gait assessment protocol. The GAITRite 6 m walkway system was used in the earlier study, and the 1 m acceleration and deceleration zones were located out of the walkway range, resulting in a total of 8-metre distance, since it was postulated that a more extended walkway is linked to a faster gait speed with more distance for acceleration [41]. Like the abovementioned phenomenon with habitual gait speed, the differences in results are explicable, particularly in the healthy older participants [37,42]. 

Normative references can be a valuable benchmark and goal-setting tool in primary care and health promotion settings. Gait speeds serve as a vital sign for functional mobility assessment [1]. Gait speed screening can assess early signs of conditions affecting mobility, such as sarcopenia or frailty [6,9,10]. The predictive nature of gait speed promotes tailored health promotion initiatives and facilitates research and population health development.

### 4.2. Strengths and Limitations

Our study has several strengths. Firstly, it established a simple, reliable, and reproducible 4 m gait speed test protocol that is readily deployable to the community without requiring sophisticated equipment. Secondly, the age range of the participants was well represented, with a sizable sample from the prospective study design that recruited participants around Singapore. Thirdly, our statistical models allowed for the assessment of non-linear effects of age, height, and BMI, better representing the data from commonly used simple linear models. Lastly, the derived results are presented as an online dashboard that facilitates a comparison of the gait speed test results. The features can be beneficial for large-scale primary care or public health screening with visualisation to enhance the public’s understanding.

There are a few limitations to this study. The final sample size included for statistical analysis was six participants less than the original sample size estimation. Furthermore, the number of participants from the sub-groups of females aged 71–80, females with height ≥1.8 m, and female obesity class II were underrepresented. This study did not include the participants’ education levels during data sampling despite some studies suggesting that educational level can positively influence gait speeds [11,13]. Additionally, our protocol excluded participants requiring walking or mobility aids/assistance, presuming that these individuals would be expected to have an altered gait speed. As a result, this has partly contributed to the lower representation of the age group 71 to 80, and it is uncertain if the reported results would under- or overestimate the gait speed in this subgroup. Finally, factors such as the influence of temporal or spatial parameters and body compositions were not investigated, yet they may also predict gait speeds. Future studies should consider exploring such variables.

## 5. Conclusions

This study determined a good-to-excellent reliability of the dynamic 4 m gait speed test protocol, implying the feasibility and deployment of simple gait speed assessment in community or public health settings without sophisticated testing equipment. The established gait speed normative references for ages 21 to 80 hold substantial merit in establishing performance benchmarks and guiding primary care interventions and public health strategies. Gait speed is a versatile tool that can enhance the quality of care in primary care settings and promote health and well-being across the lifespan. Further interventional and longitudinal studies should explore other variables given the multifactorial nature of gait speed influences and understand changes in gait speed over time and its association with health outcomes, utilisation, and costs.

## Figures and Tables

**Table 1:** Reliability measures of the 4 m gait speed tests.

Reliability Test	4 m Habitual Gait Speed Test ICC (95% CI)	4 m Fast Gait Speed Test ICC (95% CI)
Inter-Rater	0.99 (0.98 to 0.99)	0.99 (0.97 to 0.99)
Intra-Rater		
Rater 1	0.93 (0.85 to 0.96)	0.87 (0.73 to 0.93)
Rater 2	0.92 (0.84 to 0.96)	0.90 (0.80 to 0.95)
Rater 3	0.93 (0.85 to 0.97)	0.93 (0.86 to 0.97)
Rater 4	0.95 (0.89 to 0.97)	0.90 (0.81 to 0.95)
Test–Retest	0.87 (0.85 to 0.90)	0.84 (0.80 to 0.87)

Notes. ICC: intraclass correlation coefficient; CI: confidence interval.

**Table 2:** Demographics of the study participants.

Demographics	Total	Males	Females	*p*-Value
Participants, n	379	178	201	
Age, years (IQR)	45.0 (26.0 to 59.0)	44.0 (IQR 26.0 to 58.0)	49.0 (IQR 26.0 to 59.0)	0.730
Height, m (IQR)	1.64 (1.58 to 1.70)	1.71(1.67 to 1.75)	1.59 (1.55 to 1.62)	<0.001
Weight, kg (IQR)	62.0 (54.6 to 70.3)	68.8 (63.0 to 76.0)	56.0 (51.0 to 62.7)	<0.001
BMI, kg/m^2^ (IQR)	22.9 (20.8 to 25.3)	23.4 (21.6 to 25.7)	22.0 (20.2 to 24.3)	<0.001
First habitual gait speed, m/s (IQR)	1.08 (0.97 to 1.23)	1.08 (0.97 to 1.19)	1.08 (0.96 to 1.25)	0.800
95% CI	1.05 to 1.11	1.05 to 1.11	1.04 to 1.12	
Second habitual gait speed (m/s)	1.09 (0.96 to 1.22)	1.09 (0.98 to 1.21)	1.10 (0.95 to 1.23)	0.700
95% CI	1.06 to 1.11	1.05 to 1.12	1.04 to 1.12	
First habitual gait speed–second habitual gait speed, m/s (IQR)	0.00 (−0.50 to 0.05)	−0.00 (−0.05 to 0.04)	0.00 (−0.04 to 0.06)	0.320
95% CI	−0.01 to 0.01	−0.02 to 0.01	−0.01 to 0.01	
Average habitual gait speed, m/s (IQR)	1.08 (0.97 to 1.22)	1.09 (0.98 to 1.21)	1.08 (0.95 to 1.23)	0.870
95% CI	1.06 to 1.11	1.05 to 1.12	1.04 to 1.12	
First fast gait speed, m/s (IQR)	1.54 (1.40 to 1.71)	1.56 (1.42 to 1.73)	1.52 (1.38 to 1.68)	0.080
95% CI	1.51 to 1.57	1.52 to 1.64	1.49 to 1.56	
Second fast gait speed, m/s (IQR)	1.55 (1.40 to 1.72)	1.56 (1.40 to 1.78)	1.54 (1.39 to 1.68)	0.100
95% CI	1.51 to 1.58	1.51 to 1.65	1.48 to 1.59	
First fast gait speed–second fast gait speed, m/s (IQR)	0.01 (−0.06 to 0.06)	0.01 (−0.06 to 0.06)	0.01 (−0.07 to 0.06)	0.850
95% CI	−0.01 to 0.02	0.03 to 0.02	−0.02 to 0.03	
Average fast gait speed, m/s (IQR)	1.55 (1.40 to 1.70)	1.57 (1.41 to 1.77)	1.54 (1.39 to 1.67)	0.100
95% CI	1.52 to 1.59	1.51 to 1.63	1.39 to 1.67	

Values are expressed as the median and interquartile range (IQR); n: number of participants; *p* < 0.05 represents a significant value; m: metres; kg: kilogram; BMI: body mass index; m/s: metres/second; 95% CI: 95% confidence interval.

**Table 3:** Mean gait speed by age, height, and BMI categories, stratified by gender.

Group	Habitual Speed				*p* Value	Fast Speed				*p* Value
	n	Male	n	Female		n	Male	n	Female	
Age, years (IQR)										
21–30	63	1.14 (1.02 to 1.23)	57	1.16 (1.04 to 1.27)	0.49	63	1.72 (1.52 to 1.91)	57	1.56 (1.53 to 1.75)	0.29
31–40	19	1.13 (1.01 to 1.20)	25	1.08 (0.95 to 1.22)	0.38	19	1.67 (1.51 to 1.82)	25	1.50 (1.41 to 1.63)	0.01 *
41–50	27	1.05 (0.99 to 1.10)	27	1.09 (0.98 to 1.25)	0.28	27	1.54 (1.42 to 1.76)	27	1.57 (1.39 to 1.71)	0.80
51–60	34	1.08 (0.94 to 1.23)	49	1.03 (0.94 to 1.24)	0.79	34	1.49 (1.38 to 1.64)	49	1.60 (1.39 to 1.70)	0.31
61–70	22	1.03 (0.90 to 1.19)	35	1.02 (0.90 to 1.20)	0.91	22	1.58 (1.28 to 1.76)	35	1.52 (1.38 to 1.62)	0.53
71–80	13	1.02 (0.94 to 1.27)	8	0.99 (0.86 to 1.09)	0.51	13	1.57 (1.39 to 1.80)	8	1.41 (1.27 to 1.60)	0.12
Height, m										
<1.60	10	0.96 (0.95 to 1.11)	106	1.07 (0.95 to 1.23)	0.91	10	1.38 (1.33 to 1.68)	106	1.54 (1.39 to 1.66)	0.78
1.60–1.69	61	1.07 (0.98 to 1.19)	88	1.09 (0.96 to 1.49)	0.53	61	1.54 (1.39 to 1.78)	88	1.54 (1.37 to 1.68)	0.55
1.70–1.79	91	1.09 (0.97 to 1.21)	7	0.98 (0.94 to 1.02)	0.32	91	1.56 (1.44 to 1.75)	7	1.82 (1.43 to 2.20)	0.62
≥1.80	16	1.15 (1.05 to 1.29)	0	NA	NA	16	1.68 (1.57 to 1.80)	0	NA	NA
BMI										
Underweight (<18.5 kg/m^2^)	14	0.93 (0.76 to 1.30)	24	1.10 (1.02 to 1.25)	0.34	14	1.47 (1.41 to 1.58)	24	1.41 (1.37 to 1.57)	0.46
Normal weight (18.5–22.9 kg/m^2^)	68	1.08 (1.00 to 1.21)	96	1.10 (0.96 to 1.28)	0.92	68	1.67 (1.40 to 1.81)	96	1.55 (1.41 to 1.69)	0.26
Overweight (23.0–24.9 kg/m^2^)	40	1.11 (0.97 to 1.24)	42	1.06 (0.93 to 1.18)	0.19	40	1.58 (1.47 to 1.73)	42	1.54 (1.38 to 1.69)	0.13
Obesity class I (25.0–29.9 kg/m^2^)	42	1.08 (0.97 to 1.21)	39	1.03 (0.94 to 1.21)	0.47	42	1.56 (1.44 to 1.81)	39	1.53 (1.36 to 1.68)	0.44
Obesity class II (≥30.0 kg/m^2^)	14	1.07 (0.95 to 1.15)	0	NA	NA	14	1.44 (1.30 to 1.69)	0	NA	NA

Values are expressed as median and interquartile range (IQR); n: number of participants; m: metres; kg: kilograms. * denotes statistical significance.

**Table 4:** Univariate correlation coefficients (r) for habitual and fast gait speed and participant variables (n = 379).

Variable	Habitual Speed			FAST Speed		
	r	95% CI	*p*-Value	r	95% CI	*p*-Value
Gender	0.01	−0.01 to 0.11	0.871	0.10	−0.02 to 0.02	0.100
Age, years	−0.24	−0.33 to −0.14	<0.001 *	−0.19	0.02 to 0.03	0.002 *
Height, m	0.05	−0.06 to 0.15	0.343	0.14	0.02 to 0.03	0.023 *
Weight, kg	−0.04	−0.14 to 0.07	0.473	0.04	−0.08 to 0.16	0.487
BMI, kg/m^2^	−0.10	−0.21 to −0.000	0.043 *	−0.05	−0.17 to 0.07	0.421

n: number of participants; 95% CI: 95% confidence interval; m: metres; kg: kilogram; * denotes statistical significance.

## Data Availability

The datasets generated and/or analysed during the current study are available from MTY upon reasonable request.
